# Adaptive immune responses mediated age-related *Plasmodium yoelii 17XL* and *17XNL* infections in 4 and 8-week-old BALB/c mice

**DOI:** 10.1186/s12865-020-00391-8

**Published:** 2021-01-11

**Authors:** Qiu-bo Wang, Yun-ting Du, Fei Liu, Xiao-dan Sun, Xun Sun, Guang Chen, Wei Pang, Ya-Ming Cao

**Affiliations:** 1 Department of Immunology, College of Basic Medical Sciences, China Medical University, No.77 Puhe Road, Shenyang North New Area, Shenyang, 110122 China; 2 Department of Clinical Laboratory, Wuxi 9th Affiliated Hospital of Soochow University, No. 999 Liang Xi Road, Binhu District, Wuxi, 214000 China; 3 Department of Laboratory Medicine, Cancer Hospital of China Medical University, Liaoning Cancer Hospital & Institute, NO. 44 Xiaoheyan Road, Dadong District, Shenyang, 110042 China; 4 Department of Basic Medical Sciences, Taizhou University Medical School, No 1139 Shifu Road, Jiaojiang District, Taizhou, 317700 China

**Keywords:** Age-related, *Plasmodium yoelii 17XL*, *Plasmodium yoelii 17XNL*, Adaptive immune responses, 4-week-old BALB/c mice, 8-week-old BALB/c mice

## Abstract

**Backgroud:**

It is important to expound the opposite clinical outcomes between children and adulthood for eradicate malaria. There remains unknown about the correlation between adaptive immune response and age-related in malaria.

**Methods:**

4 and 8-week-old mice were used to mimic children and adulthood, respectively. Parasitemia and the survival rate were monitored. The proportion and function of Th1 and Th2 cells were detected by FACS. The levels of IFN-γ, IL-4, total IgG, IgG1, IgG2a and *Plasmodium yoelii* MSP-1-specific IgG were measured by ELISA.

**Results:**

The adult group showed greater resistance to *P. yoelii 17XL* infection, with lower parasitemia. Compared with 4-week-old mice, the percentage of CD4^+^T-bet^+^IFN-γ^+^ Th1 cells as well as IFN-γ production were significantly increased on day 5 p.i. in the 8-week-old mice after *P. yoelii 17XNL* infection. The percentage of CD4^+^GATA3^+^IL-4^+^ Th2 cells and CD4^+^CXCR5^+^ Tfh cells, and IL-4 production in the 8-week-old mice significantly increased on day 5 and day 10 after *P. yoelii 17XNL* infection. Notably, the levels of total IgG, IgG1, IgG2a and *P. yoelii* MSP-1-specific IgG were also significantly increased in the 8-week-old mice. PD-1, a marker of exhaustion, was up-regulated on CD4^+^ or activated CD4^+^ T cells in the 8-week-old mice as compared to the 4-week-old group.

**Conclusions:**

Thus, we consider that enhanced cellular and humoral adaptive immunity might contribute to rapid clearance of malaria among adults, likely in a PD-1-dependent manner due to induction of CD4^+^ T cells exhaustion in *P. yoelii 17XNL* infected 8-week-old mice.

## Background

Malaria is a serious infectious disease, which cause of mortality and morbidity in tropical countries [1]. According to WHO report, malaria transmission was found in 91 countries,with Africa experiencing disproportionately high malaria cases (90% of the total) and accounting for 91% of total malaria deaths worldwide [2]. Notably, children under the age of 5 years are particularly vulnerable to *plasmodium* infection. More than two-thirds of malaria deaths (70%) occur in this age group [3]. Of the five *Plasmodium* species that infect humans, *Plasmodium falciparum* and *Plasmodium vivax* are the most common, and *P. falciparum* is the most virulent and responsible for the majority of deaths [2, 3]. In addition, the multiplicity of infection (MOI) varies depending on the overall prevalence of infection in the population, and the age of the individual [4, 5]. The young children are highly susceptible to clinical illness and high parasitemia, whereas the adults are highly resistant [4], resulting in a major difference in the spectrum of disease manifestations between children and adults [6]. Therefore, understanding the immunological mechanisms involved in susceptibility to different virulent *Plasmodium* species during infection in children or adulthood could contribute to the development of an immunologically based control strategy to prevent or treat this devastating disease.

Upon infection, anti-parasite immunity plays a pivotal role in removing the parasite from the blood. Firstly innate immunity is activated via complement system, innate lymphoid cells and dendritic cells (DCs), act to limit the acute phase of parasitemia, but are insufficient to clear the infection [7–9]. When DCs present the processed antigen, adaptive immunity is activated. Direct cell cytotoxicity, cytokine secretion as well as anti-malarial antibody work together for effective parasite clearance [10–13]. Childhoods and young children are more susceptible to malaria infection than adults worldwide [4]. Age-related changes in immune systems increased prevalence of asthma, nasal polyps and lung injury [14, 15]. However, whether differences in cellular and humoral immunity lead to this age-related infection profile remains unknown. Therefore, we used different virulent *Plasmodium* (lethal *P.y17XL* and non-lethal *P.y17XNL*) strains to infect4-week-old and 8-week-old BALB/c mice to mimic infancy and adulthood, respectively, in order to characterize the relationship between immune cell responses and age-related malaria infection among different age groups, and understand the mechanism of malaria immunity. We propose that the dynamics of MOI can be explained by a model of increasing acquired immunity to blood-stage infection with age.

## Results

### Comparison of different species of Plasmodium infection course in 4-week-old and 8-week-old BALB/c mice

**Fig. 1:**
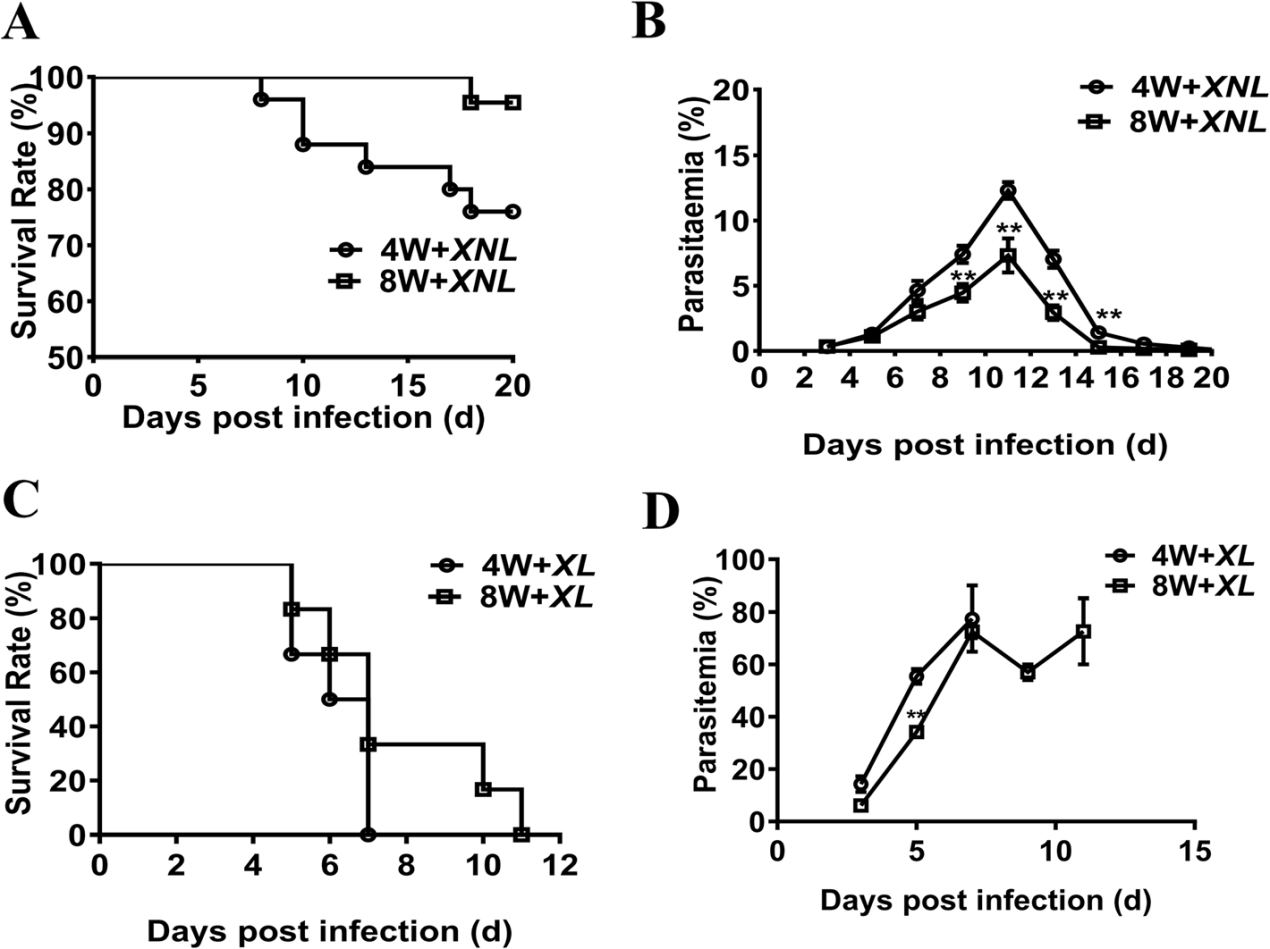
Parasitemia (**a**, **c**) and survival rate (**b**, **d**) of *Py17.XNL* or *Py17.XL* infection in 4-week-old and 8-week-old BALB/c mice. Parasitemia was calculated by counting the number of parasite-infected erythrocytes per 1000 erythrocytes. Mortality was checked daily and Mantel-Cox test analyzed the difference of survival rate (Fig. A χ2 = 3.580, *p* = 0.059, by Mantel-Cox test, Fig. C χ2 = 1.483, *p* = 0.22, by Mantel-Cox test). The data are representative of two separate experiments

To investigate the relationship between age-related host immunity against malaria infection, we used BALB/c mice of different age groups to mimic infancy and adulthood, and monitored parasitemia and the survival rate at different time points after lethal *P.y17XL* and non-lethal *P.y17XNL* infections. Within 20 days after *P.y17XNL* infection, 96% of the 8-week-old mice successfully survived whereas only 78% of the 4-week-old mice survived (Fig. 1a). In accordance with the survival rate, the parasitemia peaked at 12% in the 4-week-old mice on day 11 p.i. while it was only 7% in the 8-week-old group, although the onset of parasitemia was similar in both groups on day 3 p.i. (Fig. 1b). Similarly, parasitemia peaked at 80% in the 4-week-old mice on day 8 p.i., and all mice died; however, in the 8-week-old group, parasitemia peaked at 75% on day 8 p.i., subsequently declined, and all mice died on day 11 p.i. (Fig. 1d). Therefore, children were more susceptible to parasite infection, whereas the adult group seemed to be relatively resistant.

### Comparison of Th1 immune response in different species of Plasmodium-infected 4-week-old and 8-week-old BALB/c mice

**Fig. 2:**
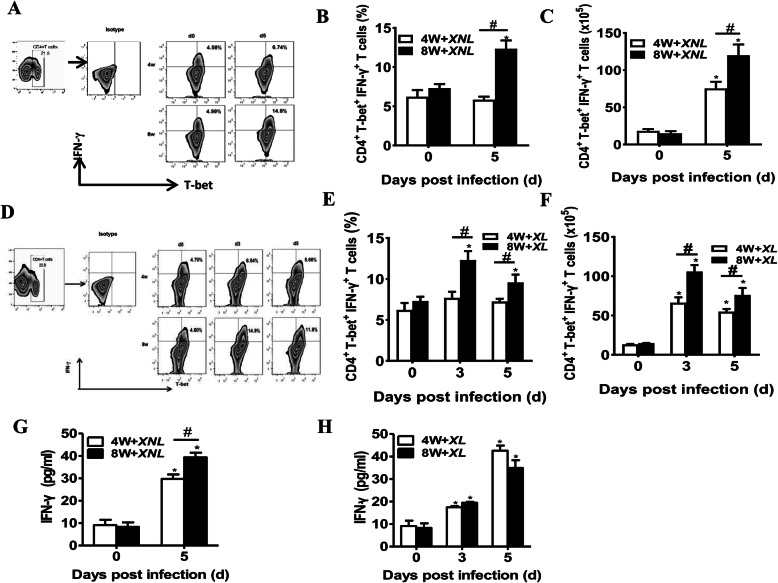
Flow cytometric and ELISA analysis demonstrated Th1 immune response in different species of Plasmodium-infected 4-week-old and 8-week-old BALB/c mice. Two-dimensional contour map (upper-left panel) (**a**), column diagram: upper-left panel, the proportion of Th1 cells in CD4^+^ T cells (**B**) and absolute cell number (**c**) of CD4^+^T-bet^+^IFN-γ^+^ Th1 cells in 4-week-old and 8-week-old BALB/c mice after *Py17XL* infection are displayed; Representative dot plots (lower-left panel) (**d**), column diagram:lower-left panel, the proportion of Th1 cells in CD4^+^ T cells (**e**) and absolute cell number (**f**) of CD4^+^T-bet^+^IFN-γ^+^ Th1 cells in 4-week-old and 8-week-old BALB/c mice after *Py17XNL* infection are displayed. Level of IFN-γ (**g**, **h**) in spleen cell supernatants in *Py17XNL/Py17XL*-infected BALB/c mice were measured. Results are presented as arithmetic mean of 9 mice each group ± SE. Single asterisk (*) and double asterisks (**) indicate *p* < 0.05 and *p* < 0.01, respectively, as compared with control mice. A single pound sign (#) and double pound sign (##) indicate *p* < 0.05 and *p* < 0.01, respectively, as compared with 8-week-old mice. Normal control group: 0 day group

Next, the relationship between Th1 cell responses and age during the early stage of plamodium infection was determined. The percentage of CD4+ T-bet+ IFN-γ + Th1 cells was determined by flow cytometry, and the level of IFN-γ in splenocytes was measured by ELISA. Compared with 4-week-old mice, the frequency and absolute number of Th1 cells were significantly increased in *P.y17XNL*-infected 8-week-old mice on day 5 p.i. (Fig. 2a-c) (*p* < 0.05). The level of IFN-γ in *P.y17XNL*-infected 8-week-old mice on day 5 p.i. had the same trend as Th1 cells (Fig. 2g). Interestingly, in *P.y17XL*-infected 8-week-old mice, the frequency and absolute number of Th1 cells peaked on day 3 p.i. (Fig. 2d-f) (*p* < 0.05), then subsequently decreased, but remained higher than normal control on day 5 p.i. (*p* < 0.05). Notably, the level of IFN-γ in lethal *P.y17XL*-infected mice was significantly increased on day 5 p.i. (*p* < 0.05), but there was no obvious difference between the 4-week-old mice and 8-week-old mice (Fig. 2h). This data suggested that enhanced Th1 cell responses might be associated with age-related non-lethal *P.y17XNL* infection and resistance during the early stage of lethal *P.y17XL* infection.

### Comparison of Th2 immune response in different species of Plasmodium-infected 4-week-old and 8-week-old BALB/c mice

**Fig. 3:**
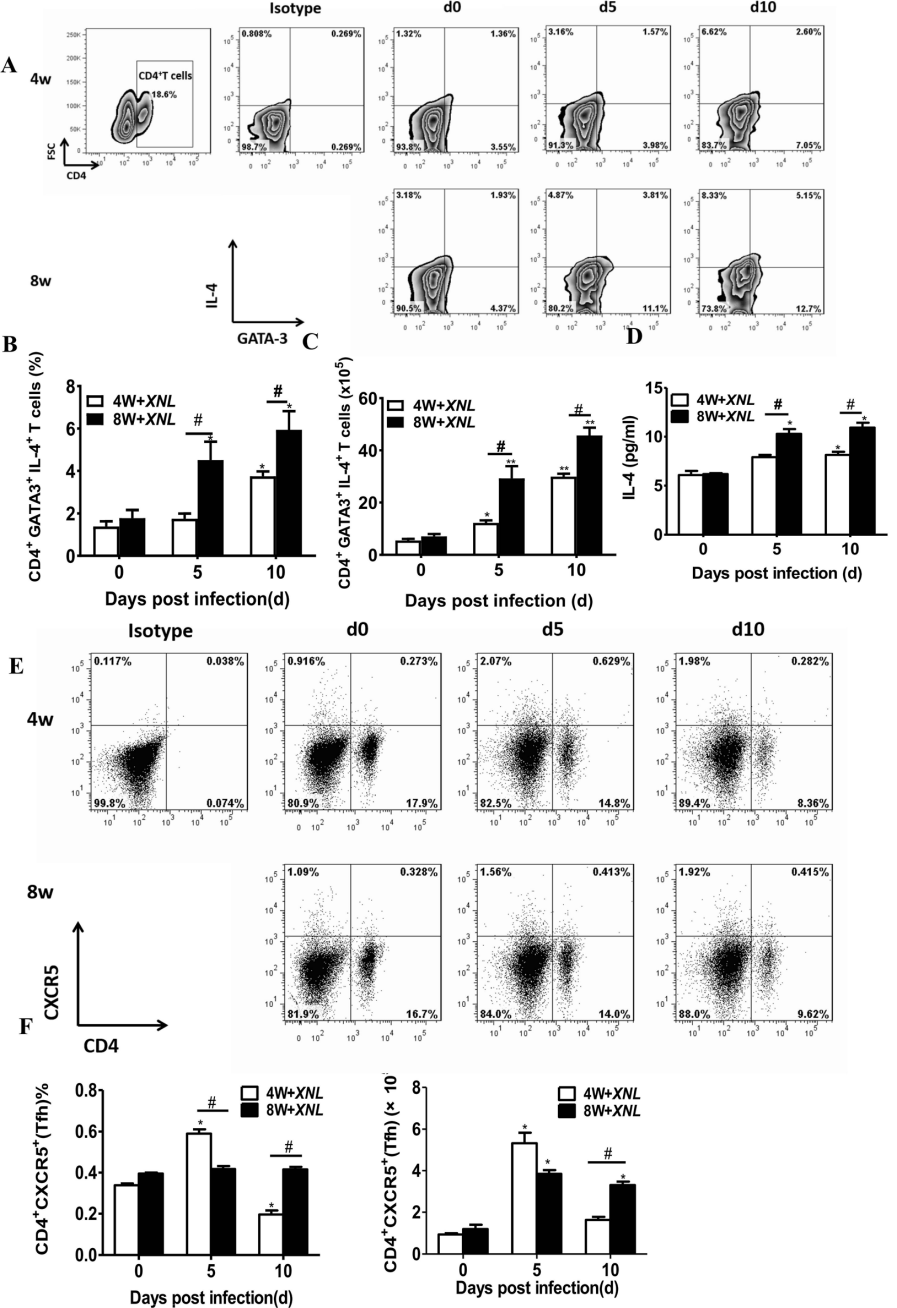
Flow cytometric and ELISA analysis demonstrated Th2 immune response in different species of Plasmodium-infected 4-week-old and 8-week-old BALB/c mice. Representative dot plots (upper-left panel) (**a**), column diagram:upper-left panel, the proportion of Th2 cells in CD4^+^ T cells (**b**) and absolute cell number (**c**) of CD4^+^GATA3^+^IL-4^+^ Th2 cells in 4-week-old and 8-week-old BALB/c mice after *P.y17XNL* infection are displayed. Level of IL-4 (**d**) in spleen cell supernatants in *P.y17XNL*-infected BALB/c mice were measured. Representative dot plots (upper-left panel) (**e**), The proportion (**f**) and absolute cell number (**g**) of CD4^+^CXCR5^+^Tfh cells were measured by flow cytometry in 4-week-old and 8-week-old BALB/c mice after *Py17XNL* infection. Results are presented as arithmetic mean of 9 mice each group ± SE. Single asterisk (*) and double asterisks (**) indicate *p* < 0.05 and *p* < 0.01, respectively, as compared with control mice. A single pound sign (#) and double pound sign (##) indicate *p* < 0.05 and *p* < 0.01, respectively, as compared with 8-week-old mice. Normal control group: 0 day group

To assess the characteristics of Th2 cell responses and their relationship with age during the late stage of plasmodium infection, we evaluated the percentage and absolute number of CD4^+^GATA3^+^IL-4^+^ Th2 cells and interleukin-4 (IL-4) production. The proportion and absolute number of Th2 cells were elevated in both groups on day 5 p.i. and day 10 p.i. after *P.y17XNL* infection as compared to normal control (Fig. 3a-c) (*p* < 0.05). Following, we found that there was obvious difference in the percentage and absolute number of Th2 cells on day 5 and 10 p.i. between the 4-week-old and 8 week-old BALB/c mice (Fig. 3b) (*p* < 0.05). Consistently, the level of IL-4 production in *P.y17XNL*-infected 8-week-old mice was significantly increased as compared to the 4-week-old mice on day 5 p.i. and day 10 p.i. (Fig. 3d) (*p* < 0.05). In addition, we detected the percentage and absolute number of CD4^+^CXCR5^+^ Tfh cells, recognized as specificized providers of cognate B cell help. The percentage and absolute number of CD4^+^CXCR5^+^ Tfh cells peaked on day 5 p.i., and then decreased to normal level on day 10 p.i. in the 4-week-old mice. However, in the 8-week-old mice, the percentage and absolute number of CD4^+^CXCR5^+^ Tfh cells were significantly increased on day 10 p.i. as compared to the 4-week-old mice (Fig. 3e, f, 3G) (*p* < 0.05). As expected, the percentage and absolute number of Th2 cells, and CD4^+^CXCR5^+^ Tfh cells, and the level of IL-4 were significantly elevated in *P.y17XNL-*infected 8-week-old group during the late stage of malaria infection. These results indicated that an enhanced Th2 immunity during non-lethal *P.y17XNL* infection might contribute to rapid clearance of *Plasmodium* in adults.

### PD-1 signal regulated immune response in different species of Plasmodium-infected 4-week-old and 8-week-old BALB/c mice

**Fig. 4:**
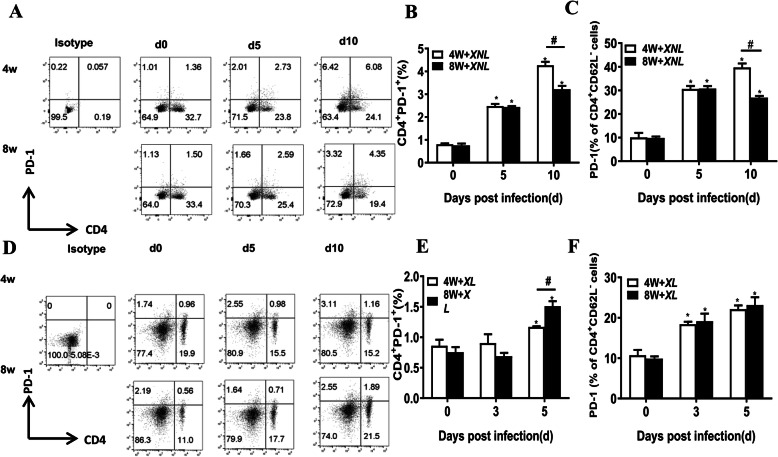
Flow cytometric analysis demonstrated PD-1 signaling promoted immune response in different species of Plasmodium-infected 4-week-old and 8-week-old BALB/c mice. Representative dot plots (upper-left panel) (**a**) and column diagram:upper-left panel, the proportion of CD4^+^PD-1^+^ cells in splenocytes (**b**) and absolute cell number (**c**) of CD4^+^PD-1^+^ cells in 4-week-old and 8-week-old BALB/c mice after *P.y17XNL* infection are displayed. Representative dot plots (lower-left panel) (**d**) and column diagram:lower-left panel, the proportion of CD4^+^PD-1^+^ cells in splenocytes (**e**) and absolute cell number) (**f**) of CD4^+^PD-1^+^ cells in 4-week-old and 8-week-old BALB/c mice after *P.y17XL* infection are displayed. Results are presented as arithmetic mean of 9 mice each group ± SE. Single asterisk (*) and double asterisks (**) indicate *p* < 0.05 and *p* < 0.01, respectively, as compared with control mice. A single pound sign (#) and double pound sign (##) indicate *p* < 0.05 and *p* < 0.01, respectively, as compared with 8-week-old mice. Normal control group: 0 day group

PD-1 signaling plays an essential role in regulating immune cell exhaustion. To explore whether PD-1 signaling mediated effector T cell exhaustion and facilitated persistent infection in infancy or adulthood, we detected the expression of PD-1 on CD4^+^ or activated CD4^+^ T cells after lethal *P.y17XL* and non-lethal *P.y17XNL* infection by flow cytometry. The expression of PD-1 on CD4^+^ or activated CD4^+^ T cells was significantly increased on day 5 and day 10 p.i. after lethal *P.y17XL* and non-lethal *P.y17XNL* infections. Compared with 8-week-old mice, the expression of PD-1 on CD4^+^ or activated CD4^+^ T cells after non-lethal *P.y17XNL* infection on day 10 p.i. was significantly raised in the 4-week-old mice (Fig. 4a-c) (*p* < 0.05). Interestingly, in lethal *P.y17XL* infection, the expression of PD-1 on CD4^+^ or activated CD4^+^ T cells was higher in the 8-week-old mice than in the 4-week-old mice on day 10 p.i. (Fig. 4d-f).

### The levels of total and P.y MSP-1-specific antibody in P.y17XNL-infected 4-week-old and 8-week-old BALB/c mice

**Fig. 5:**
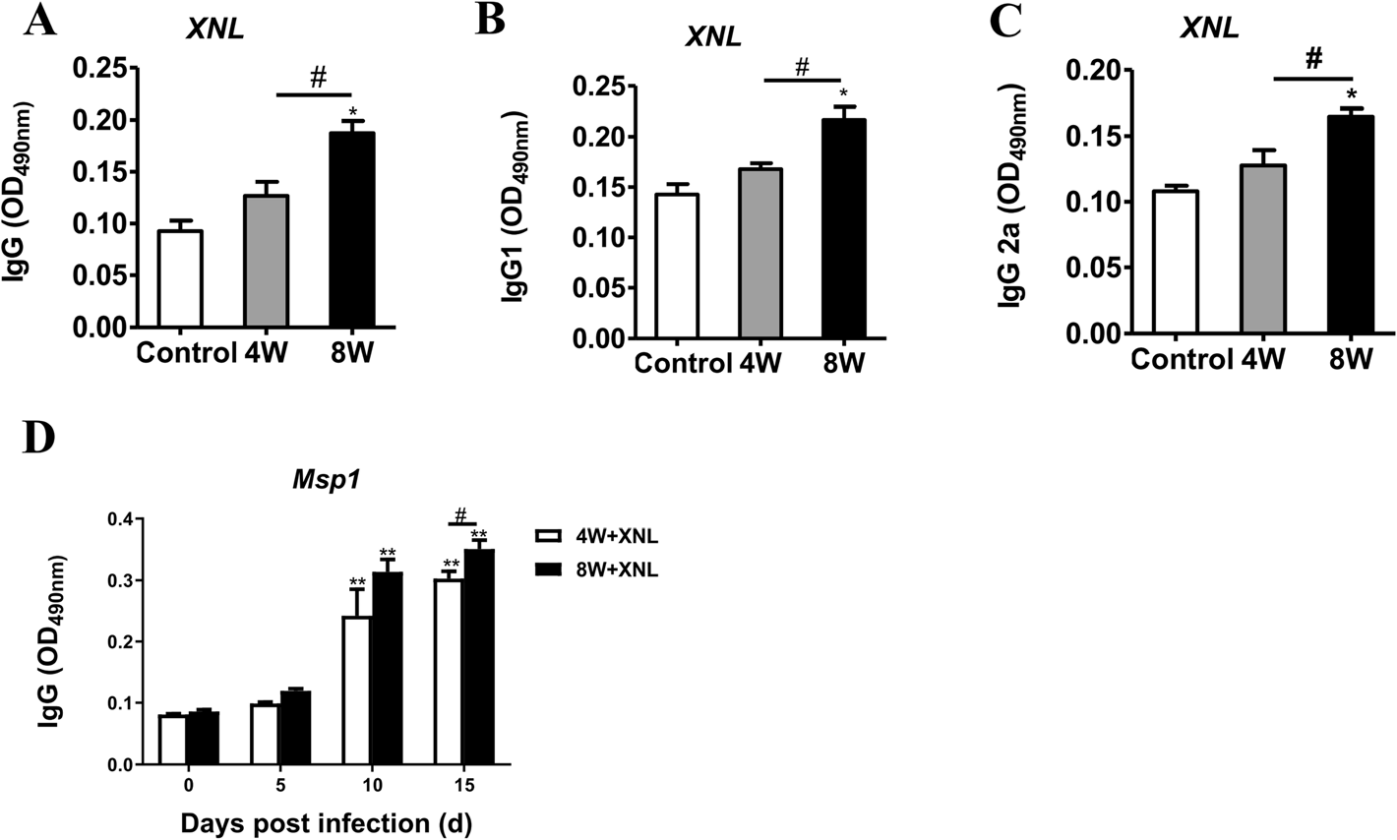
ELISA analysis demonstrated the levels of total and *P.y* MSP-1-specific antibodies in spleen supernatants of *P.y17XNL*-infected 4-week-old and 8-week-old BALB/c mice. IgG (**a**), IgG1 (**b**), IgG2a (**c**) and *P.y* MSP-1-specific IgG (**d**) were measured in supernatants of *P.y17XNL*-infected 4-week-old and 8-week-old BALB/c mice by ELISA. Results are presented as arithmetic mean of 9 mice each group ± SE. Single asterisk (*) and double asterisks (**) indicate *p* < 0.05 and *p* < 0.01, respectively, as compared with control mice. A single pound sign (#) and double pound sign (##) indicate *p* < 0.05 and *p* < 0.01, respectively, as compared with 8-week-old mice. Normal control group: 0 day group

Protection from clinical malaria has been reported to be associated with both the breadth and magnitude of the antibody responses to merozoite antigens [16]. ELISA of B cell-related total IgG, IgG1 and IgG2a also showed a significant difference in antibody production in adult mice as compared to children mice on day 10 p.i. (Fig. 5a, b, c). Interestingly, compared with 4-week-old mice, *P.y* MSP-1-specific IgG antibody production was increased in the 8-week-old mice during malaria infection (Fig. 5d) (*p* < 0.05).

## Discussion

Malaria infection is known to be age-related, with children being more susceptible than adults [17–20]. This study aimed to investigate whether the susceptibility to malaria infection in children and adulthood is associated with cellular and humoral immune responses, using a mouse model of lethal *P.y17XL* and non-lethal *P.y17XNL* infections in different age groups. Children mice were found to be more susceptible to *P.y17XNL* infection, with higher parasitemia at various time points. The adult group was more resistant to *P.y17XL* infection with lower parasitemia during the early stage of malaria infection. Importantly, enhanced cellular and humoral immunity, especially MSP-1 specific antibody, might contribute to rapid clearance of malaria in the adult group.

Malaria infections have various clinical symptoms, including febrile, anemia, acidosis and end-organ failure. To be mentioned, the difference of clinical phenotypic correlated with parasite proliferation rates, which can be controled by erythrocyte and hemoglobin polymorphisms [21]. In addition, disease profile can be determined by the strain and host [22]. In this study, 4-week-old and 8-week-old mice were used to mimic infancy and adulthood, respectively. We successfully established the age-related malaria infection mouse model to study the age-related anti-malaria immunity. Compared with 8-week-old group, the survival rate and parasitemia at different time points indicated that the 4-week-old group was same to both lethal and non-lethal parasite infections. After non-lethal *P.y17XNL* infection, parasitemia was significantly higher in the 4-week-old mice than the 8-week-old mice during the acute and chronic stages of infection. After lethal *P.y17XL* infection, a significant difference in parasitemia was observed in the early stage of infection. In accordance with the parasitemia, enhanced Th1 immune responses were only observed in the early stage in adult mice after lethal *P.y17XL* infection and enhanced adaptive immune responses (Th1 and Th2) were detected in adult mice during non-lethal *P.y17XNL* infection. These data suggested that the difference in response to non-lethal and lethal *Plasmodium* infections was associated with the pattern of immune cell responses in the host. Thus, clinical phenotypes of malaria infections can be determined by age and immune states from host.

Similar to other infectious diseases, accumulating evidences have indicated that CD4^+^ T cells are essential to control malaria infection [23–26]. Numerous studies have highlighted the role of Th1/Th2 cells or related signaling mechanisms in controlling malaria infection [27–31]. In this study, enhanced Th1 and Th2 responses were displayed in 8-week-old mice after malaria infection. Significantly higher percentage of Th1/Th2 cells and level of IFN-γ/IL-4 were observed in the 8-week-old mice as compared to the 4-week-old mice. In vitro studies also showed an enhanced Th1 cell response, which indicated an important role of Th1/Th2 cell-mediated age-related anti-malarial response. However, many studies suggested a shift from Th2 to Th1 cell responses with age. Li et al. found that IFN-γ level increased with age but not Th-related transcription factors, while IL-4 expression in plasma and CD4^+^ splenocytes declined with age [32]. A shift from Th2 towards Th1 immune responses was also observed in children with tertian or tropical malaria infection [33]. These studies partly supported our conclusion that enhanced Th1 cells might contribute to malaria clearance during the early stage of plasmodium infection. However, we observed enhanced Th2 cells during the late stage/chronic stage of malaria infection. Further studies are needed to investigate if any shift exists during the early stage of malaria infection. In addition, follicular T helper (Tfh) cells are essential for *Plasmodium* infection clearance by activating germinal center B cell responses [34–37]. Relative research found that the preferential localization of Tfh cells in the germinal center (GC) suggests a unique, intimate relationship between the Tfh cell and the B cell. Cytokines and cell-surface receptors provided by Tfh cells, as a kind of auxillary signal incompletely to keep GC B cells alive and proliferation via CD40L, IL-21 and IL-4 help [38, 39]. In this study, the percentage and absolute number of CD4^+^CXCR5^+^ Tfh cells peaked on day 5 p.i., and then decreased to normal level on day 10 p.i. in the 4-week-old mice. However, in the 8-week-old mice, the percentage and absolute number of CD4^+^CXCR5^+^ Tfh cells were significantly increased on day 10 p.i. as compared to the 4-week-old mice (Fig. 3e, f). Relative studies found that the addition of Tfh cells induces GC collapse, result for damage of B cells. Thus we speculated that GC perhaps has collapsed in young mice during the early stage of plasmodium infection, because Tfh cells increased rapidly. In addition, the GC is the primary site of B cell affinity maturation [39]. These studies supported our findings that the impaired function of antibody-secreting B cells and Tfh cells in childhood and children may account for their susceptibility to malaria infection.

We also observed a dampening of PD-1 signaling on activated CD4^+^ T cells after non-lethal *P.y17XNL* infection but not lethal *P.y17XL* infection in the 8-week-old mice. PD-1 co-inhibitory signaling was reported to regulate helper T cell differentiation and anti-*Plasmodium* humoral immunity [39], and PD-1 deficiency could enhance humoral immunity during malaria infection [40]. PD-1 was also a marker of T-cell exhaustion [41]. Several studies have also proven that chronic malaria infection drives T cell exhaustion through PD-1 signaling [42, 43]. Therefore, we speculated that during non-lethal infection, humoral immunity plays an essential role in the late stage of malaria clearance, perhaps correlated with enhanced PD-1 signaling on activated CD4^+^ T cells, which may help to drive CD4^+^ effector T cell exhaustion and promote persistent infection in children. Therefore, differences in PD-1 signaling could be observed in different age groups after non-lethal but not lethal malaria infection.

Several studies have confirmed that immune effector mechanisms are required to eliminate malarial parasites, and B cells secrete specific antibodies supported by Th2 cells, which can effectively remove the parasites to prevent the recidivation and recrudescence [44, 45]. Similarly, infusion of malaria hyperimmune serum resulted in rapid clearance of parasitized erythrocytes [45]. Merozoites proliferation from RBCs can be prevented by Anti-*Plasmodium* antibodies, depended on blocking cytoadherence to endotheliar capillary of iRBCs and promoting phagocytosis by mononuclear cells [46–48]. However, researchers found that levels of antimalarial antibodies continue to increase significantly resulting from chronic exposure to infection [49], perhaps correlated with impaired establishment of B cell memory [50]. Thus in young children,we can found the short-lived antibody responses [51–54]. In this study, we detected the levels of B cell-related total IgG, IgG1 and IgG2a in *P.y17XNL*-infected BALB/c mice. The results showed a difference in antibody production between adult and children mice, and the levels of total antibody might contribute to rapid clearance of malarial parasites in the adult group during the chronic stage of non-lethal *P.y17XNL* infection. Moreover, IgG1 and IgG3 antibodies against merozoite surface proteins (MSPs) are thought to be instrumental in protection, which is considered as a major vaccine candidate [55]. Therefore, we detected the levels of *P.y* MSP-1 specific antibody. Consistently, the dynamics of *P.y* MSP-1 specific antibody was the same as total antibody. These data implied that an enhanced antibody response during chronic stage of non-lethal *P.y17XNL* infection might contribute to rapid clearance of malaria in the adult group.

## Conclusion

Taken together, the findings of this study revealed that in non-lethal *P.y17XNL* infection, higher burden of parasitemia in children mice were associated with weakened Th1 cellular immune responses, down-regulated humoral immunity with decreased percentage and number of Th2 and Tfh cells as well as lower level of antibody secretion and enhanced PD-1 signaling on activated CD4^+^ T cells. Higher resistance to lethal *P.y17XL* infection in the early stage in adult mice was associated with enhanced Th1 cellular immune responses and weakened PD-1 signaling on activated CD4^+^ T cells. These results provide a new insight on immune responses in malaria infection.

However, we have to consider a question: the expression of PD-1 on activated CD4^+^ T cells induced depletion of immune cells. On the one hand, depletion of immune cells induced down-regulation of anti-malaria immune response; on the other hand, exhaust of immune cells reduces the immunopathological injury in malaria-infected hosts. It is very difficult to regulate the dynamic balance between them. So there are many questions to deal with about using PD-1 antagonists to treat malaria infections.

## Methods

### Mice, parasite and experimental infection

The 4-week-old (90 mice) and 8-week-old (90 mice) female BALB/c mice were purchased from Beijing Animal Institute. *P.y*17XL and *P.y*17XNL strains were provided by Dr. Motomi Torii (Department of Molecular Parasitology, Ehime University Graduate School of Medicine, Ehime, Japan). Infections were initiated by intraperitoneal (i.p.) injection of 1 × 10^6^ *P.y* 17XL or 1 × 10^6^ *P.y* 17XNL parasitized erythrocytes in BALB/c mice. All animal procedures were conducted in compliance with the Regulations for the Administration of Affairs Concerning Experimental Animals (1988.11.1), and humanely treated. The experimental mice were matched for age and sex. Parasitemia was examined by light microscopy of Giemsa-stained, tail blood smears. Mortality was monitored daily. All experiments were performed in compliance with local animal ethics committee requirements. The animals were not submitted to euthanasia during the process of *plasmodium* infection. Other mice were submitted to euthanasia during detecting the relative index in indicated time points, the way to do it is posterior cervical dislocation after eyeball blood extraction.

### Spleen cell culture

Spleen cell culture was prepared as previously described [56]. Briefly, we aseptically removed spleen from each mouse, and then passed through a sterile fine-wire mesh with 10 ml of RPMI1640 including 5% heat-inactivated fetal calf serum (FCS) (Hyclone Laboratories, Inc.), 25 mM Hepes (Life Technologies), 0.12% gentamicin (Schering, Montreal, Quebec, Canada) and 2 mM glutamine (Life Technologies). Cell suspensions were centrifuged at 350×*g* for 10 min at room temperature (RT). Using cold 0.17 M NH_4_Cl to lysed Erythrocytes. Following the cells were washed twice with fresh medium, and then viability of the spleen cells was confirmed by trypan blue exclusion, and was always > 90%. Spleen cells were adjusted to a final concentration of 10^7^cells/ml in RPMI1640 supplemented with 10% heat-inactivated FCS. Aliquots (500 μl/well) of the cell suspension were incubated in 24-well flat-bottom culture plates (FALCON) in triplicate for 48 h at 37 °C in a humidified 5% CO_2_ incubator. Then, the plates were centrifuged at 350×*g* for 10 min at RT, supernatants were collected and stored at − 80 °C until they were assayed for the levels of IFN-g, IL-4, IgG, IgG1, IgG2a and *P. y* MSP-1-specific IgG.

### Cytokine analysis

Commercial enzyme-linked immunosorbent assay (ELISA) kit smeasured levels of IFN-γ and IL-4 according to the manufacturer’s protocols (R&D Systems, Minneapolis, MN). Using a microplate reader read the OD values at 450 nm. The concentrations of cytokines in samples were calculated against the standard curve generated using recombinant IFN-g and IL-4, respectively.

### Multiplex assay for antibody determination

Levels of total serum IgG, IgG1, IgG2a and *P.y* MSP-1-specific IgG were measured by ELISA as previously described with some modifications [57]. Briefly, Maxisorp flat-bottomed, 96-well microplates were coated overnight at 4 °C with 50 μg of *P.y* MSP-1 antigens in a carbonate-bicarbonate buffer (pH 9.6). The plates were washed with PBS-Tween (PBS-T) and blocked with 0.05% bovine serum albumin (BSA)-PBS-T. Next, 100 μl of plasma dilutions in 0.05% BSA-PBS-T (1:50 for *P.y* MSP-1 IgG) were added in duplicate and incubated at RT for 2 h. After washing with PBS-T, the plates were incubated with horseradish peroxidase-conjugated goat anti-mouse IgG (Sigma, USA) at a dilution of 1:5000. The OD values were read in a microplate reader at 490 nm.

### Cell surface/intracytoplasmicstaining and flow cytometry

To assess the function of CD4^+^ T cells, we detected Tfh (CD4^+^CXCR5^+^cells), CD4^+^PD-1^+^cells and CD4^+^CD62^−^PD-1^+^cells, spleen cells from BALB/c mice infected with *P.y17XL/P.y17XNL* at different time points were double-stained with FITC-conjugated anti-CD4 (clone GK1.5, BD), BV421-conjugated anti-PD-1 (clone J43, BD), PE-conjugated anti-CXCR-5 (clone 2G8, BD) and APC-conjugated anti-CD62L (MEL-14, BD), followed by two washes, staining and analysis by flow cytometry.

To assess dynamics of Th1(CD4^+^T-bet^+^IFN-γ^+^) cells and Th2 (CD4^+^GATA3^+^IL-4^+^) cells, spleen cells from BALB/c mice infected with *P.y17XL/P.y17XNL* at different time points were triple-stained with fluorescein isothiocyanate (FITC)-conjugated anti-CD4 (clone GK1.5), PE-conjugated anti-T-bet (clone eBio4B10, eBioscience), APC-conjugated anti-IFN-γ (XMG1.2, BD) for Th1 cells, and FITC-conjugated anti-CD4 (clone GK1.5), PE-conjugated anti-GATA-3 (clone L50–823, BD), APC-conjugated anti-IL-4 (clone 11B11, BD) for Th2 cells. After stimulation for 2 h with PMA and ionomycin at 37 °C, Golgi Stop (BD Bioscience) was added to each reaction (1:500, vol/vol). After co-culture for 4 h at 37 °C, the cells were washed with 3% FCS and then resuspended in 100 μl of 3% FCS. FITC-anti-CD4, PE-anti-T-bet and PE-anti-GATA3 were added for surface staining. Then, the cells were fixed and permeabilized, and intracytoplasmic staining was performed using allophycocyanin (APC)-anti-IFN-γ. We used the isotype control antibodies as follows: Table 1. All antibodies were purchased from BD Pharmingen.

**Table 1:** Information of all isotype control antibodies

Antibodies	Iostype antibodies
FITC-conjugated anti-CD4 (clone GK1.5, BD)	FITC Rat IgG2a, κ Isotype Control,BD
BV421-conjugated anti-PD-1 (clone J43, BD)	BV421 Hamster IgG2, κ Isotype Control,BD
PE-conjugated anti-CXCR-5(clone 2G8, BD)	PE Rat IgG2a, κ Isotype Control,BD
APC-conjugated anti-CD62L (MEL-14, BD)	APC Rat IgG2a κ Isotype Control,BD
PE-conjugated anti-T-bet (clone eBio4B10, eBioscience)	PE Mouse IgG1 κ Isotype Control,eBioscience
APC-conjugated anti-IFN-γ (XMG1.2, BD)	APC Rat IgG1, κ Isotype Control,BD
PE-conjugated anti-GATA-3 (clone L50-823, BD)	PE Rat IgG2b κ Isotype Control,eBioscience
APC-conjugated anti-IL-4 (clone 11B11, BD)	APC Rat IgG1, κ Isotype Control,BD

### Statistical analysis

All analyses were performed using GraphPad Prism version 6.0 (GraphPad Software, La Jolla, CA). Data are presented as mean ± standard error of the mean (SEM). Survival analysis was performed using the Kaplan-Meier log-rank test. Statistical significance of differences between the two groups was assessed by unpaired Student’s t-tests. *P*-values were calibrated using Bonferroni correction, and were considered statistically significant if they were less than 0.05.

## Data Availability

The datasets used and/or analyzed during the current study are available from the corresponding author on reasonable request.

## Abbreviations

*P.y17XL*: *Plasmodium yoelii 17XL*
*P.y17XNL*: *Plasmodium yoelii 17XNL*
DCs: Dendritic cells
IL-4: Interleukin-4
Tfh: Follicular T helper cells
RBCs: Red blood cells
iRBCs: infected RBCs
MSPs: Merozoite surface proteins
i.p.: intraperitoneal
FCS: Fetal calf serum
